# Nonlinear dynamics based machine learning: Utilizing dynamics-based flexibility of nonlinear circuits to implement different functions

**DOI:** 10.1371/journal.pone.0228534

**Published:** 2020-03-03

**Authors:** Behnam Kia, Allen Mendes, Akshay Parnami, Robin George, Kenneth Mobley, William L. Ditto

**Affiliations:** 1 Nonlinear Artificial Intelligence Laboratory, Department of Physics, North Carolina State University, Raleigh, North Carolina, United States of America; 2 First Pass Engineering, Castle Rock, Colorado, United States of America; University of the West of England, UNITED KINGDOM

## Abstract

The core element of machine learning is a flexible, universal function approximator that can be trained and fit into the data. One of the main challenges in modern machine learning is to understand the role of nonlinearity and complexity in these universal function approximators. In this research, we focus on nonlinear complex systems, and show their capability in representation and learning of different functions. Complex nonlinear dynamics and chaos naturally yield an almost infinite diversity of dynamical behaviors and functions. Physical, biological and engineered systems can utilize this diversity to implement adaptive, robust behaviors and operations. A nonlinear dynamical system can be considered as an embodiment of a collection of different possible behaviors or functions, from which different behaviors or functions can be chosen as a response to different conditions or problems. This process of selection can be manual in the sense that one can manually pick and choose the right function through directly setting parameters. Alternatively, we can automate the process and allow the system itself *learn* how to do it. This creates an approach to machine learning, wherein the nonlinear dynamics *represents* and *embodies* different possible functions, and it *learns* through training how to pick the right function from this function space. We report on how we utilized nonlinear dynamics and chaos to design and fabricate nonlinear dynamics based, morphable hardware in silicon as a physical embodiment for different possible functions. We demonstrate how this flexible, morphable hardware learns through learning and searching algorithms such as genetic algorithm to implement different desired functions. In this approach, we combine two powerful natural and biological phenomenon, Darwinian evolution *and* nonlinear dynamics and chaos, as a dynamics-oriented approach to designing intelligent, adaptive systems with applications. Nonlinear dynamics embodies different functions at the hardware level, while an evolutionary method is utilized in order to find the parameters to implement the right function.

## Introduction

The human brain is the most complex, sophisticated system yet known to us, and nonlinear dynamics play a crucial role in the brain and in the way the brain operates, processes information, and learns [1,2,3]. To engineer any system that emulates, simulates, or imitates the brain’s functionality, there usually must be some form of nonlinearity built into the system. For example, a nonlinear dynamics-based cognitive prosthesis was designed to restore long-term memory [4]. In another example a nonlinear activation function is a fundamental element used by multilayer neural networks and deep learning [5] to obtain a universal function approximator [6]. In neuromorphic engineering, where the goal is to design and fabricate a brain-like system on silicon, neurons are highly nonlinear elements [7].

The main premise and advantage of nonlinear dynamics is that a simple nonlinear system is fully capable of exhibiting diverse, complex behaviors. Living systems by utilizing nonlinear dynamics can exhibit diverse and complex behaviors [8]. These systems can explore many different behaviors or reactions that their nonlinearity provides to them and adaptively select the ones that best meet their needs and conditions. Langton investigated this subject in context of computation in his seminar paper titled “Computation at the edge of chaos: Phase transitions and emergent computation [9],” and many others followed his footsteps [10].

There is a basic and fundamental connection between nonlinear dynamics and computation, intelligence, and learning. We are exploring such a connection through the design and fabrication of a nonlinear dynamics based morphable silicon circuitry. Our main approach towards achieving computation and learning is that 1) nonlinear dynamics provides diversity in terms of different types of functions the system can implement (through containing many different dynamical patterns) *and* morphability (through controls) which creates a suitable mechanism for plasticity and learning in which we can pick and choose different functions from the available set of functions. 2) Our system is fundamentally implemented at the hardware level as opposed to fundamentally software-based learning methods. In nature there is no software, it is the physical organism itself that shows learning and intelligence, and the intelligence is intertwined with the dynamics and physics of the organism. These two hypotheses combined propose that we need a nonlinear dynamics-based hardware that provides flexibility and plasticity *at the hardware level* as a platform for learning and intelligence.

It is important to note that neuromorphic engineering follows a similar path in the sense that its focus is to design and fabricate neurons and neural networks in silicon with the hope that the fabricated nonlinear hardware will exhibit brain-like behavior and intelligence. However, the difference between our approach and neuromorphic engineering is that neuromorphic engineering stays faithful to the dynamics of neurons and tries to replicate their behavior, whereas we pick and choose the right type and amount of nonlinearity (and chaos) and utilize it for everyday applications.

There are also multiple other reported dynamics-based approaches to machine learning and artificial intelligence. One approach is called reservoir computing [11], where complex dynamics provides universality–the capability of a system to implement any other function from a specific class. Our approach is similar to such dynamics-based approaches to designing a universal function approximator but with the addition that we have instantiated the complex dynamics through the design and fabrication of nonlinear circuits.

Recently, we designed and fabricated an integrated circuit for a nonlinear dynamics-based morphable logic block in which the complex nonlinear dynamics of a transistor circuit is utilized to implement reprogrammability and morphability [12]. The very same circuit was capable of implementing all different two-input, one-output digital functions due to the flexibility contributed by its inherent nonlinearity. The fabricated nonlinear circuit could be instantly reprogrammed by providing different digital control inputs in order to implement different digital functions. These programing inputs changed the bifurcation parameters of the nonlinear circuit, or perturbed the initial condition of the nonlinear circuit, and therefore changed the behavior of the circuit in a controlled manner and the function that it implements [12].

A full description of this chaos-based approach to computing has been previously reported [12,13]. However, the main idea is briefly reviewed here. A dynamical system maps its initial state to future states, so one can observe and consider it as a function that maps inputs (the initial state) to the outputs (the final state). In this context, the inherent dynamics performs the inputs-outputs mapping, meaning that the functions are dynamically implemented. Therefore, we call it *dynamics-based computing*. A nonlinear dynamical system, such as a nonlinear circuit, has a complex, flexible dynamics, containing many different behaviors and as a result it can be morphed to implement many different functions. In a nonlinear dynamical system, there are parameters called bifurcation parameters that change the qualitative behavior of the circuit. By changing these parameters, one can alter the dynamics of the circuit, and therefore the type of functions that it can implement. Furthermore, when a nonlinear system is in a chaotic regime, it is very sensitive to its initial state or initial conditions. A change to its initial state can change the future state, and as a result, the type of function that it builds. The number of such different functions that a nonlinear, complex system can implement exponentially increases by the evolution time [14]. In a discrete-time dynamical system, evolution time is the number of iterations that the dynamical system takes before producing the output, whereas in a continuous-time dynamical system the evolution time is the time interval during which the system evolves from the initial condition and produces the outputs. This observation is compatible with the basic dynamics of nonlinear complex systems, where nearby orbits diverge and behave very differently as they evolve over time. In a perfect world, where there is no noise or other limiting factors, the number of such distinct functions that a nonlinear complex system can implement exponentially and boundlessly increases. But in a real, practical world, and in the presence of noise and other nonidealities, just a limited set of such functions can be robustly obtained. Here we are focusing on implementing an array of logic blocks that each block is capable of implementing different possible two-input, one-output digital functions.

**Fig 1:**
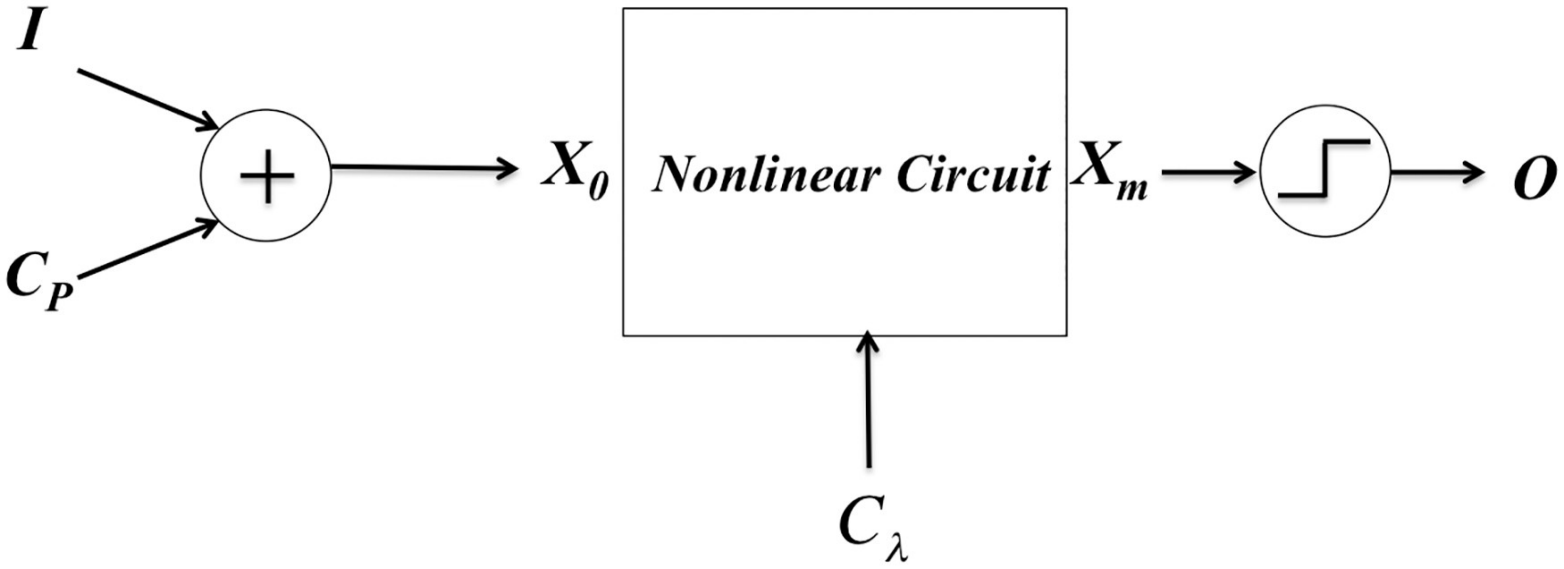
Block diagram for a NLDBM logic block.

Each nonlinear dynamics-based morphable (NLDBM) logic block in our design receives 4 binary control inputs. Two control inputs set the bifurcation value of the NLDBM logic block, and the other two perturb the initial state of the circuit. A pictorial block diagram of the logic block is shown in Fig 1. A two-bit control input, *C*_*p*_, along with the data inputs *I*, set the initial state of the nonlinear circuit. The circuit then evolves under its nonlinear dynamics. This nonlinear dynamics can be altered with two-bit bifurcation parameter, *C*_*λ*_. By using these control inputs, one can manually or adaptively morph the NLDBM logic block to implement different two-input, one-output functions [13].

In this paper we report our newest fabricated nonlinear dynamics-based morphable (NLDBM) hardware, comprised of a series of NLDBM logic blocks and show how it’s inherent circuit-level and dynamics-based morphability can provide us with a novel platform to implement representation and learning in the form of morphable hardware and evolution.

In our NLDBM hardware the control inputs (whose values are the parameters we need to learn) and the outputs are both discrete-valued, for training we use evolutionary computation such as genetics algorithm, which is easier to implement for such a discrete optimization problem.

Evolvable hardware is not a new concept. Usually evolvable hardware is implemented on a reprogrammable computing system, such as Field Programmable Gate Arrays, FPGAs [15], Field-Programmable Transistor Arrays, FPTAs [16] and field programmable analog arrays, FPAAs [17]. The intrinsic programmability and flexibility within these platforms, combined with an evolutionary computation algorithm to adjust and program the flexibility, results in an evolvable hardware that can adaptively evolve to implement a given task or adjust itself to the environment. The aim of this paper is not to introduce the concept of evolvable hardware, since evolvable hardware has been known for many years. Neither is the aim of this specific paper to solve difficult application examples that others have failed to solve. Our aim in this paper is to combine 1) NLDBM hardware and the inherent dynamics-based flexibility and universality that comes with it, with 2) biologically inspired evolution and learning, in order to obtain an intelligent, evolvable hardware and demonstrate it as a novel platform to be considered for implementing learning systems. We should note that the application examples that we present in this article are relatively simpler compared to other published evolvable hardware applications. The reason is that we designed and fabricated our own NLDBM hardware platform along with our own interface circuit, and the software. By contrast, in other conventional evolvable hardware implementations, off the shelf, commercial-grade digital reconfigurable hardware and its accompanying CAD tools that were already perfected by their manufacturers were utilized. Because of our more basic hardware/software platform, we were limited to simpler examples that we could implement on our platform as a proof of concept. However, these simple, but nontrivial examples demonstrate the main concepts of our approach and its potentials.

The main difference between conventional evolvable hardware and the dynamics-based evolvable hardware reported in this paper is that in our approach different functions coexist within the same hardware, whereas in conventional FPGAs different functions need to be loaded into the hardware. More specifically, in a NLDBM logic block, the complex dynamics of the circuit embodies different types of functions, and therefore one can dynamically and instantly pick and choose different functions by providing different sets of inputs. In sharp contrast, in conventional look-up-table based FPGAs, different functions need to be loaded to the system. This reprogramming usually requires halting the system and loading the new programing data into the look-up-tables. Learning process includes trying different sets of the control inputs, observing the outputs, and adjusting the control inputs accordingly and iterating this process until the system behavior converges to an optimal solution. As a result, when the machine learns by trying and experimenting, it is very important to the system to be able to switch from one function to another quickly. Previously we have discussed how a simple circuit contains exponentially many different functions and how easy it is switch from one function to another [12,14].

There are similarities and differences between our work and neural networks. Both systems are programmable (trainable) function approximators. Please see [18,14] for discussions on nonlinear dynamics as flexible, programmable function approximator. Both of them receive inputs, undergo some nonlinear transformation, and an output is produced from the final state. But they are different in the sense that there are no synoptic weights in our approach in order to program the system, instead we use dynamical parameters in order to alter the system and the function it implements. The focus in our approach is mostly on the nonlinear dynamics of the system and how it can implement different functions. But in conventional neural networks although nonlinearities exist in the form of nonlinear activation function, such nonlinearities and their effects on the computation is not necessarily the primary point of interest.

## Nonlinear dynamics-based morphable hardware

**Fig 2:**
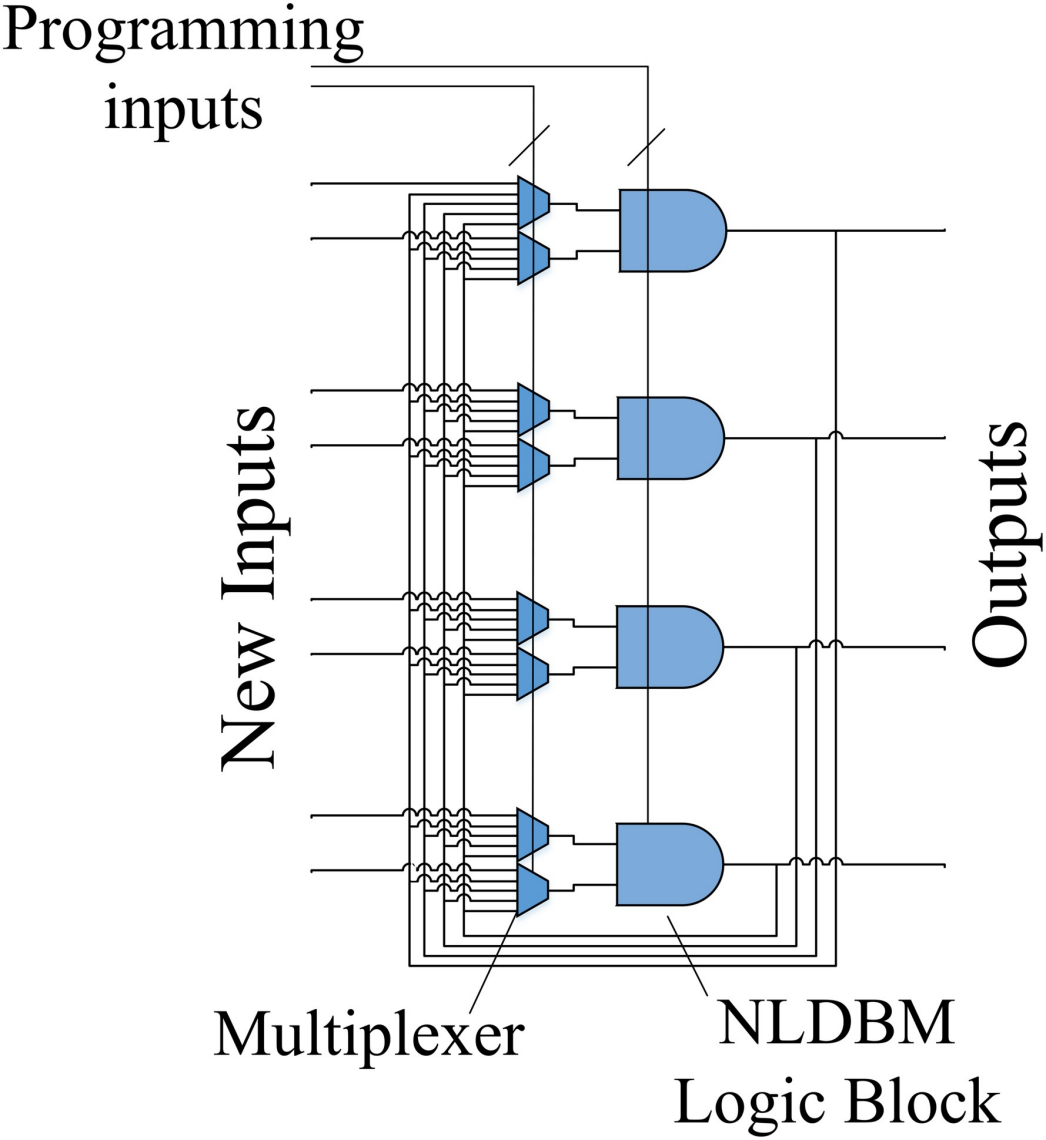
The architecture of the new NLDBM hardware.

**Fig 3:**
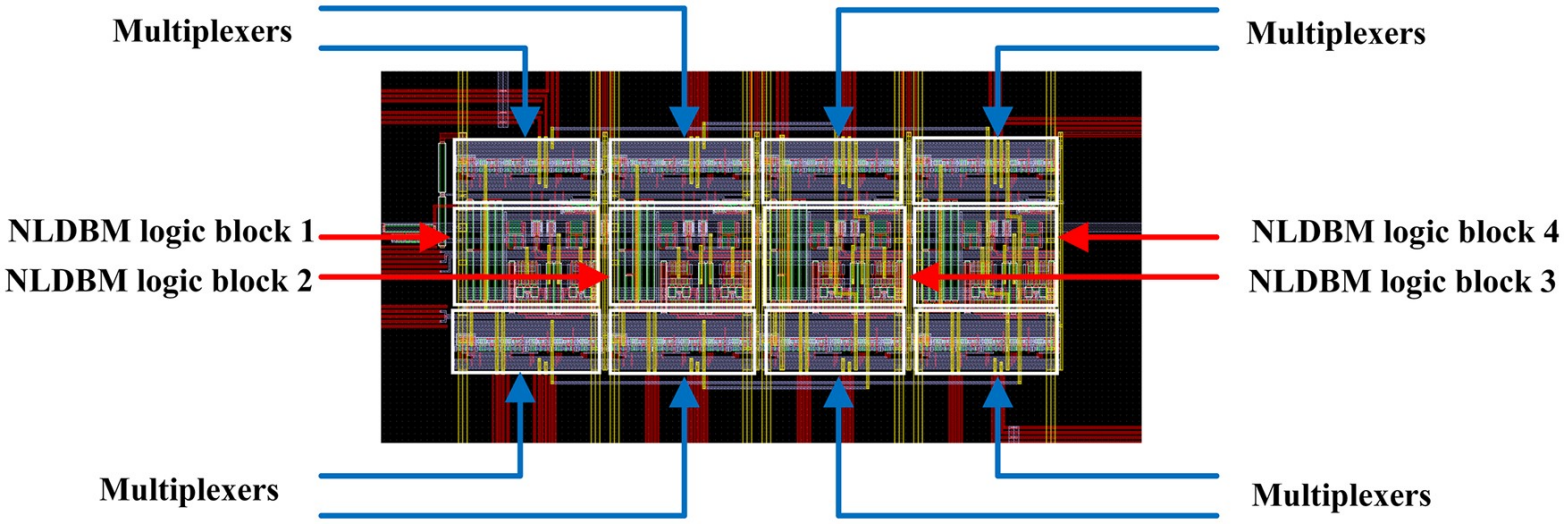
The designed layout. 4 NLDBM logic blocks and 8 multiplexers are labeled.

In this section we report our newest, latest fabricated hardware that we use as a platform for learning. We fabricated a NLDBM hardware that consists of a column of NLDBM logic blocks that can be instantly reprogrammed to implement different operations and functions. The outputs of this column of NLDBM logic blocks are fed back to the input lines of the same column, thereby enabling a single column to implement digital circuits that would otherwise require multi-column designs. The NLDBM hardware has the following architecture, shown in Fig 2. The designed layout for this architecture is presented in Fig 3.

The architecture of the new NLDBM hardware is composed of four two-input, one-output NLDBM logic blocks arranged in a column. These individual NLDBM logic blocks are the same morphable logic block that we introduced before [12], but now we have fabricated four of them in a column. Each NLDBM logic block is instantly reconfigurable. The main feature of Fig 2 architecture is that it preserves the instant reconfigurability of each NLDBM logic block, and transforms it to instant reconfigurability of the whole system, where the architecture can be instantly reprogrammed to implement higher-level functions.

These four NLDBM logic blocks in the column can receive new inputs from the input pins of the chip, or the outputs from the previous computation can be fed-back to the blocks again. There are multiplexers that can be programmed to select different inputs to each input line of each NLDBM logic block. Both the NLDBM logic blocks and the multiplexers are programmable.

By changing the programing inputs, one can program the hardware to implement different functions and behaviors. By letting the hardware itself evolve and learn how to morph and adjust its inherent flexibility, the architecture shown in Fig 2 becomes an evolvable, adaptive hardware platform that can be trained to implement different operations with no need for direct programing. The complexity of the operations the hardware can implement depends on the number of times the outputs of the hardware are fed back and used as the new inputs. Each process of feeding back the outputs to the inputs is effectively equivalent to adding a new column of logic when this hardware’s operation is unrolled over time. In this article we do not discuss the number of functions or the level of complexity of such functions that the hardware can implement. However, we bring the attention of the readers to the results of [19], where it was shown that the power of a logical circuit in implementing different functions exponentially increases by adding new columns to the circuit. In this article, we mostly focus on implementing exemplary functions in order to demonstrate how the hardware can be trained to implement different functions.

The NLDBM hardware was designed using Cadence software suite, and we used the 0.6μm, 5-V On-Semi C5 process to design the circuits, and the MOSIS service to fabricate it. The fabricated chip was placed in a 40-pin package. This specific chip packaging came with 40 pins, which put a limit on the number of data inputs, control inputs to program, and verification test pins, that we could have and access. As a result, we had to make some compromises. For example, instead of having separate bifurcation parameter inputs for different NLDBM logic block in the column, all four of them share the same control inputs. This has a limiting effect on the flexibility of the hardware but in the future, this can be overcome with more sophisticated designs as needed.

## Training the hardware

In the previous section we introduced our new NLDBM hardware, where the nonlinearity gives us flexibility and therefore a function space to choose from. In this section we focus on training such morphable hardware, in which the NLDBM hardware learns to implement a desired function. The key performance measures used in this research in order to assess the results are the reliability, consistency, and repeatability of experimental observations from the fabricated hardware. More specifically, when we train the hardware (or a model of the hardware) to implement a given function, and we obtain a set of control values that program the hardware to implement the function, we apply these control values repeatedly and observe the output of the hardware to ensure that the hardware implements the function consistently. We repeated this process tens of time, and during different hours of the day. The results that we report in this paper are checked to be repeatable, and robust to noise and room temperature variations. It is also worth to note that there is an inherent robustness built into the results obtained from evolutionary methods. If a set of chromosomes, here control values, do not result in robust behavior of the circuit, the population is not going to converge to said control values. Therefore, the population will keep evolving until it converges to a set of control values that results in consistent, reliable behavior across different members of the population.

Machine learning has been a very successful approach to artificial intelligence, in which the machine–here the NLDBM hardware–learns through examples how to implement a given task. In supervised machine learning, we have access to labeled training data. Labeled training data is a set of input, output 2-tuples. This training data tells the machine what outputs we expect it to produce for different given input data. The learner, which can be a neural network, NLDBM hardware, etc., is a parametric universal function approximator that for different parameter values implements different functions. In a neural network, the parameters are the weights and bias values, and in our NLDBM hardware the parameters are the control input values to program the NLDBM logic blocks and to connect them together through the multiplexers. Now the question of learning is reduced to *which parameter values shall we use in order to implement a desired function*. This is the place that the training data becomes handy. We are looking for a function that can fit the training data–maps the inputs to the outputs. To do so, we usually define a loss function that measures and quantifies discrepancy between the expected outputs and what the learner, here the NLDBM hardware, produces. Therefore, the problem in hand can be morphed to an optimization problem, in which we search for parameters that minimizes this cost function. Finding optimal parameters for highly nonlinear learners such as multilayer neural networks is a non-convex optimization problem, and in terms of computational complexity, it is a *hard problem* and there is no algorithm that can perform this task in polynomial time [20]. However, there are heuristic methods such as stochastic gradient descent methods or genetics algorithms that can help us to find near optimal parameters that result in satisfactory performance for these learners.

There are two important notes related to machine learning in general and our work reported in this article in particular. First, statistical learning is more than just an optimization problem. In statistical learning, we need to make sure that the learner can generalize beyond the examples in the training set, and can perform well on new, unseen data as well [21]. In this article, we are training the NLDBM hardware to implement functions that have a small set of inputs. Therefore, during training, we can give the entire input space of the problem to the learner and train it on it. As a result, generalization is not an issue for this specific type of problems because the learner observes and trains on the entire input space. But for any other problem that the hardware is trained on a sample set of the input space (not the entire input space), generalization needs to be addressed properly. The second note is more of a philosophical nature. It is a common knowledge that highly nonlinear learners such as multilayer neural networks are extremely powerful in modeling and representation, but they are hard to train [19]. As a result, for many years machine learning practitioners preferred simpler learners, in which training is a convex optimization problem, and therefore tractable to solve. But this simplicity of training came with a catch; simpler learners are much less capable than deep multilayer networks in representation and learning. The current revolution of deep learning started when researchers found a path to train very capable, but hard-to-train nonlinear multilayer neural network [22]. The point is that working with highly nonlinear learners is challenging, but extremely rewarding at the same time. As a result, nonlinearity should not repel us from studying such new models, but it should be a driving force to study more complex nonlinear systems. This has been our motivation to study and investigate NLDBM hardware for performing different computation and learning tasks.

As discussed in the introduction, training the NLDBM hardware is a discrete optimization problem, and methods such as genetics algorithm are easier to develop and apply for such problems. We follow two separate approaches described below toward implementing this evolvable NLDBM hardware.

1. Model-based learning. In this approach we use a model of NLDBM hardware, and let this model evolve and learn how to implement or solve a given function or problem.
2. Direct training of the fabricated chip. In this approach, there is no modeling involved; instead the actual fabricated NLDBM hardware is directly trained and evolved to implement the desired functions.

### Model-based learning

The first approach is model-based in the sense that we used a model of the Fig 2 architecture combined with an evolutionary computation algorithm to evolve this model to implement the desired operation or function. When the evolution is successfully finished, we take the resulting programing inputs and apply them to the fabricated NLDBM hardware in order to verify the accuracy of the results. As evolution is done externally and on a computer, this approach is much faster than the direct training method.

Our architecture is composed of four NLDBM logic blocks. Each logic block is modeled with its instruction set, which determines which function each NLDBM logic block implements when different control inputs are applied. Each NLDBM logic block receives four control bits, and can implement different two-input, one-output combinational functions. We applied different control inputs to each NLDBM logic block inside the fabricated chip and observed the association between control inputs and the type of function that the logic block implements. Table 1 is the instruction set obtained for these NLDBM logic block. The first column is the four-bit control inputs (C_1_C_2_C_3_C_4_.) to program the logic block, and the second column is the function number that the logic block implements. Two control inputs C_1_ and C_2_, are basically *C*_*p*_ in the Fig 1 block diagram, which perturb the initial state of the nonlinear circuit. Two other control inputs, C_3_ and C_4_, are the bifurcation inputs, C_λ_. This instruction set is a part of the model for the fabricated hardware. Note that all four NLDBM logic blocks have the same instruction set because they have the identical circuit design and implementation. Of course, there are some fabrication variations, but those variations are not great enough to change the instruction set and we observe the same instruction set for different instances of the logic block on the chip.

**Table 1:** The instruction set of a single fabricated NLDBM logic block.

Control bits (C_1_C_2_C_3_C_4_)	Functions
0000	13
0001	8
0010	9
0011	8
0100	5
0101	1
0110	1
0111	1
1000	13
1001	1
1010	9
1011	0
1100	5
1101	1
1110	5
1111	1

The function numbers in the second column of Table 1 instruction set is nothing more than a unique number to label and identify different functions. The function number for each function is obtained based on the outputs of the function when different combinations of the inputs are applied to the logic block. As an example, when different input combinations 00, 01, 10, and 11 are applied to the NLDBM logic block, and the outputs are, as an example, 1, 0, 1, and 0 respectively as shown in Table 2, we label the function as function number 10 because 10 is the outputs in decimal number system: 1(MSB)×2^3^+0×2^2^+1×2^1^+0(LSB)×2^0^ = 10.

**Table 2:** An example function implemented by the two-input, one-output NLDBM logic block.

I_1_	I_2_	Output
0	0	1(MSB)
0	1	0
1	0	1
1	1	0(LSB)

The rest of the model is concerned with the connections in terms of how the inputs are connected to the NLDBM logic block via multiplexers, how the outputs of the logic blocks are fed back to their inputs, and how these connections can be programmed. We also included the limitation imposed by the packaging in the model as well. Also, for simplicity, we apply the new inputs to the first column, while the other columns receive the outputs of the logic blocks from their previous columns as their inputs.

**Fig 4:**
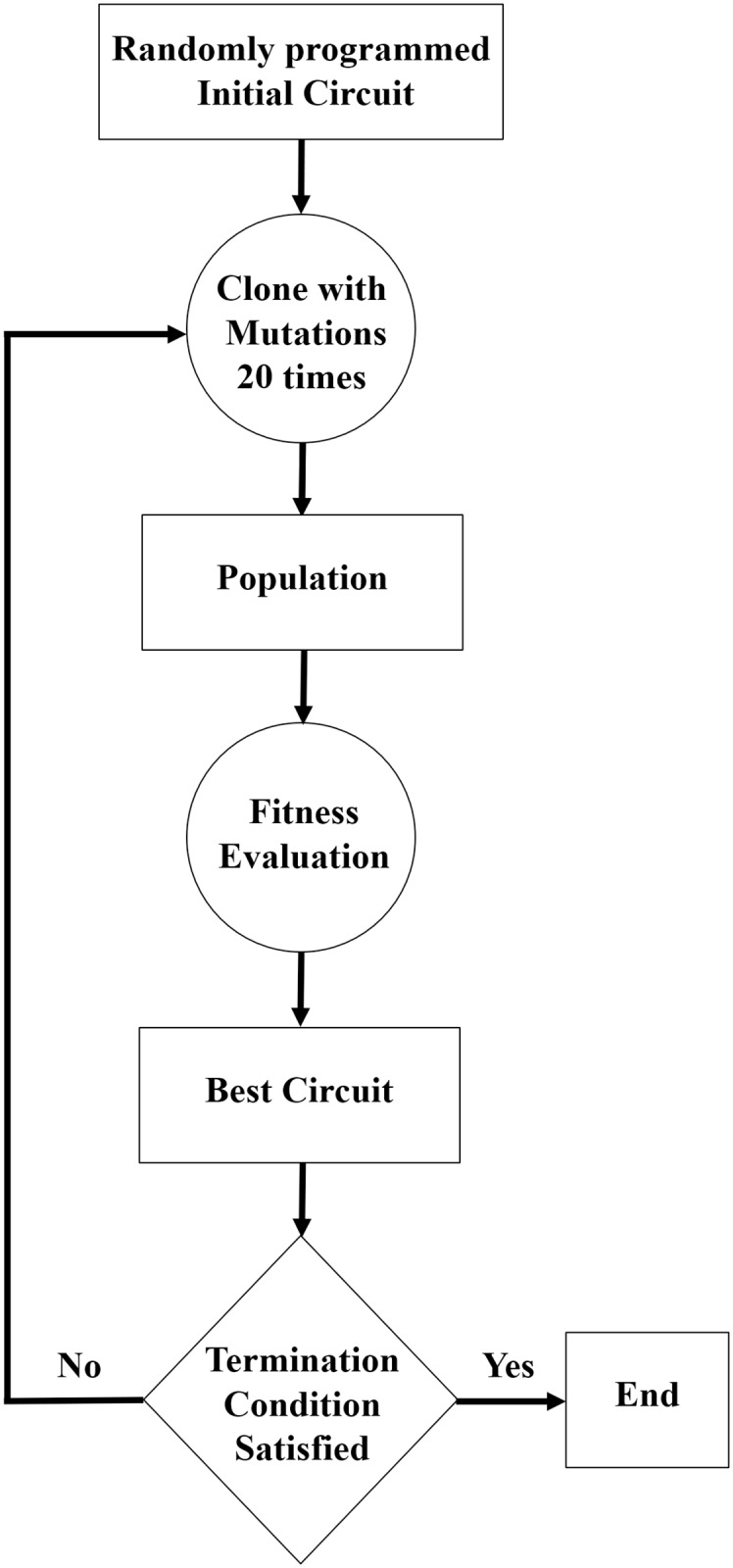
Flowchart for the genetic algorithm used in this article.

NLDBM hardware provides flexibility at the hardware level, and we use an evolutionary computation algorithm to evolve and train this inherently flexible hardware. The main idea of Darwinian evolution is that the individuals among a species that are more fitted to an environment or a given situation are more likely to survive and reproduce. Those better-fitted parents pass on their good genes to offspring comprising the next generation of species that should be more fitted to the environment than the previous generation. This type of natural selection, combined with random mutations during reproduction, provide a path for a species to evolve towards an optimal generation that has adapted to the environment or the given problem. Following this approach to optimization, different heuristic optimization and learning methods are introduced [23]. Evolutionary computation is an umbrella term that is used for different algorithms that mimics natural, Darwinian evolution. And different variations of each algorithm have been reported. The flowchart for the technique that we used in this research as a simple imitation of Darwinian evolution is depicted in Fig 4. This technique can be considered as a genetic algorithm without crossover, which has been used in evolvable hardware design before [24]. We used this technique because of its simplicity. However, we expect that with further research on application of different types of evolutionary computation or other learning methods on NLDBM hardware results can be further improved.

Consider the control inputs to the NLDBM hardware as the genotype, and the operation that the programmed NLDBM hardware implements as the phenotype. The final goal is to find specific control inputs that program the NLDBM hardware to implement the desired operation. Under the genetic algorithm, the flexible NLDBM hardware evolves and morphs in order to implement the desired operation. Here we have focused on simple, basic digital functions as objective functions that we need the hardware to implement. These functions can be presented in a truth table, and this truth table tells which outputs the function should produce for different input values. This table is basically our training dataset. Our goal is to train the NLDBM hardware to implement this table. More specifically:

1. The algorithm starts from a random population, in this case random initial control inputs. The algorithm randomly sets the programing inputs and obtains a fitness value for the resulting “individual” (programmed NLDBM hardware model) by comparing the outputs against the expected outputs—the desired truth table. With each correct output bit, the individual is awarded with one point. The final fitness value is the number of these points combined. Of course it is very unlikely that the initial random control inputs will program the morphable hardware to implement the desired truth table. Therefore, the NLDBM hardware model has to undergo evolution to perfect its performance. The most fitted random individual–the one with the highest fitness value–is found and this individual will be used as the parent in step 2.
2. A new population of offspring is obtained from the parent based on mutation. Mutation is implemented by simply flipping some of the control bits. The number of bits flipped, also known as mutation rate, changes over time from generation to generation as the individuals evolve and approach the optimal individual and finial solution. At the beginning we start from a high mutation rate (flipping a lot of bits) in order to quickly explore the solution space. But then as the population starts to converge to the final optimal solution, we reduce the mutation rate, therefore performing the final fine-tuning.
3. The most fitted offspring–the one with the highest fitness value–is found, and if its fitness value is greater or equal to the fitness value of the parent, it replaces the parent, otherwise we continue with the original parent.
4. This completes one iteration of the simple genetic algorithm. If the desired maximum fitness is achieved, here correctly implementing the entire truth table with no issue, the evolution stops and it produces the final control inputs. If not, the evolution process repeats from step 2. If the algorithm reaches the maximum and has to be restarted with some other initial values and settings from step 1.

#### Addition operation

Imagine the aim is to perform an addition operation and the size of operands is two-bit each. The truth table (the training data) for this two-bit addition is shown in Table 3.

**Table 3:** Truth table for a two-bit addition operation.

A_1_	A_0_	B_1_	B_0_	Q_2_	Q_1_	Q_0_
0	0	0	0	0	0	0
0	0	0	1	0	0	1
0	0	1	0	0	1	0
0	0	1	1	0	1	1
0	1	0	0	0	0	1
0	1	0	1	0	1	0
0	1	1	0	0	1	1
0	1	1	1	1	0	0
1	0	0	0	0	1	0
1	0	0	1	0	1	1
1	0	1	0	1	0	0
1	0	1	1	1	0	1
1	1	0	0	0	1	1
1	1	0	1	1	0	0
1	1	1	0	1	0	1
1	1	1	1	1	1	0

**Fig 5:**
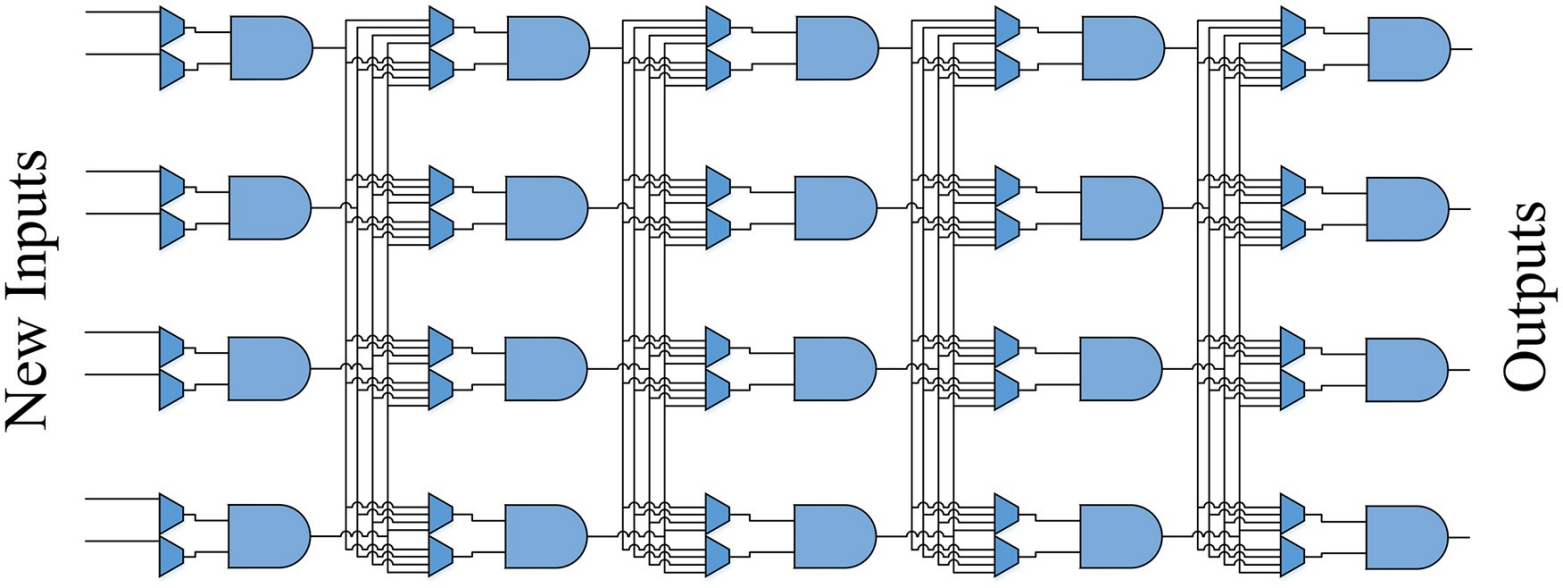
The unrolled architecture effectively equivalent to the case when the outputs of the fabricated circuit are fed back to the inputs five times.

A and B are two two-bit operands, and Q is the three-bit output. We set the number of column iteration of the final design to five. This column iteration number dictates how many times the outputs of the single-column design in Fig 2 are fed back to the input lines to perform more computation on them before producing the final outputs. When the outputs of the single-column fabricated hardware are fed back to the inputs 5 times, the result is equivalent to Fig 5 unrolled architecture, which contains five separate columns of NLDBM logic blocks. The NLDBM logic blocks can be, and usually are, programmed differently for each column. Therefore, we will have different control inputs for different columns.

After 148,844 iterations of the genetic algorithm and a population size of 20, the algorithm converges to control inputs that program the NLDBM hardware to implement the addition operation.

The genetic algorithm was relatively slow to converge and it needed 148,844 iterations to converge, when one would expect a faster convergence for an addition operation of two-bit operands. The reasons for this slow convergence were the limitations that we had imposed on the morphable hardware model due to factors such as packaging where we have two common control inputs for all logic blocks. Such constrains and limitations greatly reduced the flexibility of the fabricated NLDBM hardware and it took a longer time for the algorithm to find a set of control inputs in this constrained solution space. In our future hardware designs, where we will not have such limitations and each logic block will have its own separate control inputs, we would expect much faster convergence because the full flexibility and reconfigurability of all the logic blocks will be at our disposal.

**Fig 6:**
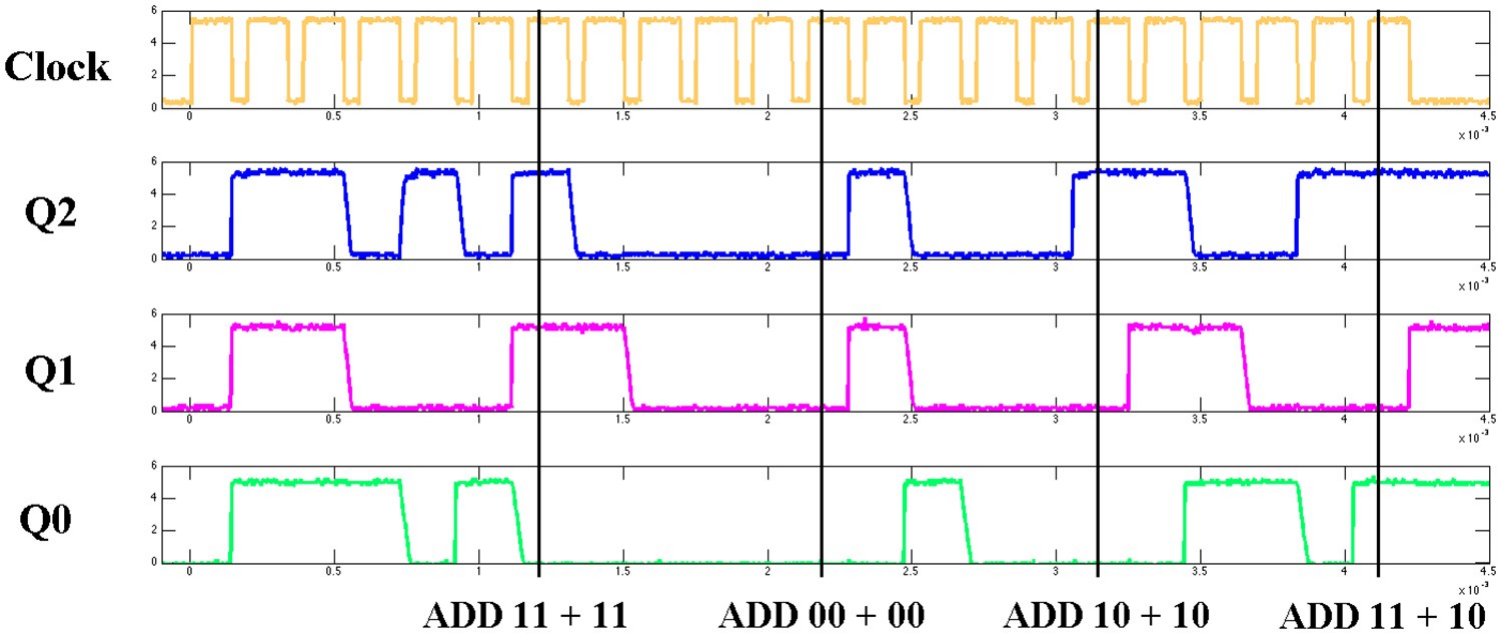
Observed output waveforms from NLDBM hardware. The results of four addition outputs can be observed from the outputs.

Once we obtained the solution from the evolved model, we confirmed the results by applying the programing inputs to the fabricated chip to test that it was performing the desired operation. As a visual example, see Fig 6, where the output waveforms of the fabricated chip are shown for four addition operations in a row. We used the results of the genetic algorithm as the control inputs, and provided four sets of two-bit binary data inputs (11, 11), (00, 00), (10,10), and (11,10) as operands. Three output bits Q_2_Q_1_Q_0_ can be read from the waveform for the different input sets as 110, 000, 001, and 101, which are the expected outputs for an addition operation. Note that each instruction requires 5 clocks, because we set the number of column iterations to 5. At each clock one iteration of the column is executed.

To ensure reliability and repeatability of the results, we applied a simple statistical test. We repeated each experiment 10 times for each set of inputs and operations and collected the results. We only accept results that are consistent across all runs of the experiment. All the results reported in this article have passed this statistical test to insure reliability and robustness of the results.

## Direct training of the fabricated chip

In this section, our fabricated hardware undergoes training to learn to implement desired functions. The genetic algorithm as the training algorithm applies different programing inputs to the physical NLDBM hardware, and obtains the real output from the fabricated hardware to determine the fitness value of the current circuit design. The example function that we use here is Hamming weight. The Hamming weight of a binary string is the number of “1”s in it. Table 4 shows three-bit streams, and we want to count the number of 1’s.

**Table 4:** Hamming weight function as an example objective function. Q_1_Q_0_ presents the number of 1’s in the A_2_A_1_A_0_ stream.

A_2_	A_1_	A_0_	Q_1_	Q_0_
0	0	0	0	0
0	0	1	0	1
0	1	0	0	1
0	1	1	1	0
1	0	0	0	1
1	0	1	1	0
1	1	0	1	0
1	1	1	1	1

Similar to our model-based learning, we made some assumptions and set some parameters manually, although we could have let the system learn them as well. We set the number of column iterations to three, assuming that three columns are enough to implement the Hamming weight of three-bit inputs. The inputs are fed to the first column, and the output of the three logic blocks from the third iteration of the column are considered to be the outputs.

We used the same previously described genetic algorithm for training, but with the important difference that now instead of using a model of the chip, the fabricated chip itself is in the learning loop, the circuit receives the data and control inputs, and the outputs are read from the output pins of the circuit. The algorithm converged after 1,240 iterations, and the NLDBM hardware was able to perform the Hamming weight calculation.

**Fig 7:**
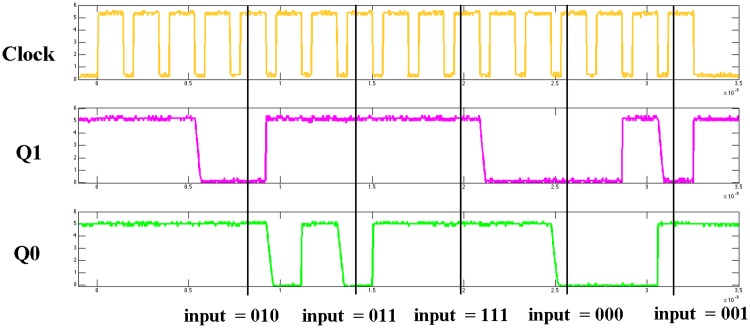
Observed output waveforms from NLDBM hardware. The hardware calculates Hamming weights of five different inputs.

As a visual demonstration, Fig 7 shows the output waveforms of the fabricated hardware for five Hamming weight calculations in a row. Five different strings of inputs are given to the hardware, and the hardware produces two-bit outputs, Q_1_Q_0_, as the output. Note that in this example each instruction is executed in 3 clocks, because we set the number of column iterations to 3.

This direct training of the NLDBM hardware itself instead of using models has interesting advantages. For example, the NLDBM hardware can adapt to internal or external imperfections or changes or faults that were not captured within the model. In our previous work [25] we demonstrated a NLDBM logic block that was overheated to a point that its operation and performance changed, but then it was manually reprogrammed to perform the same operations reliably, albeit with a different set of control inputs. That idea, in conjunction with evolvable, self-learning nonlinear hardware that we introduced in this article, results in an adaptive nonlinear hardware that can also tolerate faults and withstand variable environments and conditions.

## Conclusions

We were able to combine nonlinear dynamics and chaos theory with Darwinian evolution, two popular and powerful natural, biologically-oriented concepts, in order to design and fabricate VLSI circuitry and demonstrate a proof of concept implementation of nonlinear dynamics-based learning. We introduced our latest fabricated nonlinear dynamics-based morphable (NLDBM) hardware, which is morphable and flexible, and can be reprogrammed to implement different functions. We demonstrated how this NLDBM hardware can learn to implement different functions with no need for direct programing.

We also showed how a model of the NLDBM hardware can be used for training, and how the NLDBM hardware itself can be directly trained to evolve and learn to implement different objective functions.

The main conclusion of our paper is that nonlinear dynamics and chaos can provide a suitable platform for learning. Different functions coexist within the complex dynamics of nonlinear circuits. The system can learn how to morph in order to solve a given problem. In this context, learning happens at the circuit level by utilizing the inherent dynamics-based morphability of the nonlinear hardware.

In this paper, we have demonstrated the learning capability of the dynamics-based hardware by implementing exemplary functions. Questions such as the learning capacity of the hardware, optimal training methods to learn different functions, or generalization beyond the training dataset warrant further investigation.
